# Efflux reprogramming promotes *Staphylococcus aureus* persistence with *Pseudomonas aeruginosa* in cystic fibrosis

**DOI:** 10.1128/aac.01070-26

**Published:** 2026-10-08

**Authors:** Q. C. Truong-Bolduc, Y. Wang, B. Alvarez-Carcamo, C. Steifman, B. G. Lawton, L. M. Yonker, B. P. Hurley, L. G. Rahme, D. C. Hooper

**Affiliations:** 1 Infectious Diseases Division and Medical Services, Massachusetts General Hospital 551672, Boston, Massachusetts, USA; 2 Harvard Medical School 1811 , Boston, Massachusetts, USA; 3 Department of Pediatrics, Massachusetts General Hospital 2348, Boston, Massachusetts, USA; 4 Department of Pediatrics, University of Texas Southwestern Medical Center 196285, Dallas, Texas, USA; 5 Mucosal Immunology and Biology Research Center, Massachusetts General Hospital 2348, Boston, Massachusetts, USA; 6 Department of Surgery, Massachusetts General Hospital 548305, Boston, Massachusetts, USA; The Peter Doherty Institute for Infection and Immunity, Melbourne, Victoria, Australia

**Keywords:** *S. aureus*, efflux pump, cystic fibrosis, ASM, *P. aeruginosa*

## Abstract

*Staphylococcus aureus* persists in cystic fibrosis (CF) airways despite *in vitro* antibiotic susceptibility, suggesting that efflux reprogramming driven by environmental and polymicrobial interactions with *Pseudomonas aeruginosa* promotes survival in this setting. In this study, we investigated how CF-like conditions and co-infection with *P. aeruginosa* influence *S. aureus* physiology and antimicrobial response. Using artificial sputum medium (ASM) and co-culture models, we found that ASM induced a selective transcriptional response characterized by upregulation of the multidrug efflux pump *tet38*, while other pumps remained minimally expressed. In the presence of *P. aeruginosa*, *S. aureus* maintained *tet38* expression but additionally induced *norB*, indicating a redistribution of efflux activity under polymicrobial stress. *P. aeruginosa* metabolites, including quinolines and phenazines, contributed to this response, and deletion of *tet38* or *norB* significantly reduced *S. aureus* fitness in ASM and co-culture conditions. Analysis of *S. aureus* clinical isolates from people with CF revealed a similar hierarchy, with Tet38 and NorB as the predominant efflux pumps, particularly in *S. aureus* isolates co-recovered with *P. aeruginosa*. Despite exhibiting only low-level increases in MICs, these isolates demonstrated enhanced survival in ASM. Notably, *S. aureus* isolates paired with clinical *P. aeruginosa* strains showed improved survival compared with survival when paired with a *P. aeruginosa* reference strain, consistent with adaptation toward coexistence. Together, these findings demonstrate that CF-like environmental conditions and polymicrobial interactions drive selective reprogramming of efflux pumps that promote *S. aureus* persistence without conferring high-level resistance, highlighting a mechanism of antibiotic tolerance not captured by standard susceptibility testing.

## INTRODUCTION

Chronic airway inflammation is a defining feature of cystic fibrosis (CF) and contributes to persistent bacterial infection and progressive lung damage. CF results from mutations in the CFTR gene, leading to impaired mucociliary clearance, accumulation of thick mucus, and sustained neutrophil-dominated inflammation (1–4). Although highly effective CFTR modulator therapies have improved clinical outcomes, bacterial colonization of the airways remains common, even after CFTR modulator treatment (3, 5–7). These observations highlight a critical gap in our understanding of how pathogens persist in the CF lung despite therapeutic intervention (8).

The CF airway is a complex polymicrobial environment in which bacterial interactions play a central role in colonization, persistence, and disease progression. Common pathogens include *S. aureus* (*SA*) and *P. aeruginosa* (*PA*), which are frequently co-isolated from respiratory samples of people with CF (pwCF) (9–13). While *PA* has been shown to outcompete and kill *SA* under standard laboratory conditions (14–16), clinical and longitudinal studies indicate that these organisms often coexist for extended periods in the CF airway (17–19). Co-infection with both species is associated with more severe disease and worse clinical outcomes compared with single-species infection (20, 21). These findings suggest that interactions between *SA* and *PA* evolve *in vivo* toward coexistence, but the bacterial mechanisms that enable this persistence remain incompletely defined.

Multiple adaptive strategies have been described for *SA* survival in the presence of *PA*. These include metabolic reprogramming and the emergence of small-colony variants, which are associated with impaired respiration and increased tolerance to antibiotics and host defenses (15, 22–26). *PA* secretes a range of metabolites, including phenazines and quinolines, that disrupt *SA* respiration and growth (27, 28). In response, *SA* can alter its physiology to withstand these stresses, suggesting that interspecies interactions are a major driver of bacterial adaptation in the CF airway.

Efflux pumps are an important component of *SA* adaptation and antimicrobial response. Among the major chromosomally encoded multidrug (MDR) efflux pumps in *S. aureus* are NorA, NorB, Tet38, MepA, and MdeA. These conserved transporters form part of the core *S. aureus* efflux network, although their expression levels and relative contributions to antimicrobial adaptation vary depending on strain background and environmental conditions. NorA, NorB, MepA, and MdeA contribute to the extrusion of structurally diverse antimicrobial compounds (29, 30), whereas Tet38 additionally promotes bacterial interaction with host epithelial cells, highlighting roles beyond antibiotic resistance (17, 31). Among these systems, NorA, NorB, and Tet38 have been particularly associated with decreased susceptibility to fluoroquinolones and tetracyclines (31, 32). Although MepA and MdeA represent additional components of the MDR efflux network, their relative contributions to adaptive responses under CF-associated conditions remain less defined.

Previous studies, including our own, have shown that *PA* metabolites such as pyocyanin (PYO), phenazine-1-carboxylic acid (PCA), and 3,4-dihydroxy-2-heptylquinoline (PQS) can induce efflux pump expression in *SA* or alter *SA* resistance genetic determinants (24, 33, 34). In addition, artificial sputum medium (ASM), which mimics key features of the CF airway, has been shown to modulate *SA* gene expression (35, 36). However, it remains unknown how CF-associated environmental and polymicrobial signals reshape the hierarchy of MDR efflux pump utilization, favoring specific transporters over others, and how this adaptive reprogramming promotes antibiotic tolerance and bacterial persistence.

In this study, we investigated the response of *SA* to CF-like conditions using ASM, with and without co-culture with *PA*, and extended these findings to clinical isolates from pwCF. We show that ASM induces a selective efflux response dominated by Tet38, while the presence of *PA* leads to reprogramming of efflux activity to include NorB together with Tet38. Analysis of clinical isolates revealed a similar hierarchy, with Tet38 and NorB representing the predominant efflux pumps in CF-associated *SA*. Despite exhibiting only low-level increases in MICs, these isolates demonstrated enhanced survival in ASM, particularly under polymicrobial conditions. Furthermore, *SA* isolates co-recovered with *PA* showed improved fitness in co-culture, consistent with adaptation toward coexistence.

Together, these findings support a model in which environmental conditions characteristic of the CF airway and polymicrobial interactions drive selective efflux pump reprogramming that promotes antibiotic tolerance and bacterial persistence without conferring high-level resistance. This work provides a framework for understanding the disconnect between *in vitro* susceptibility and *in vivo* persistence of *SA* in chronic CF infections.

## RESULTS

### *S. aureus* activates MDR efflux pumps in response to CF-like stress

**Fig 1:**
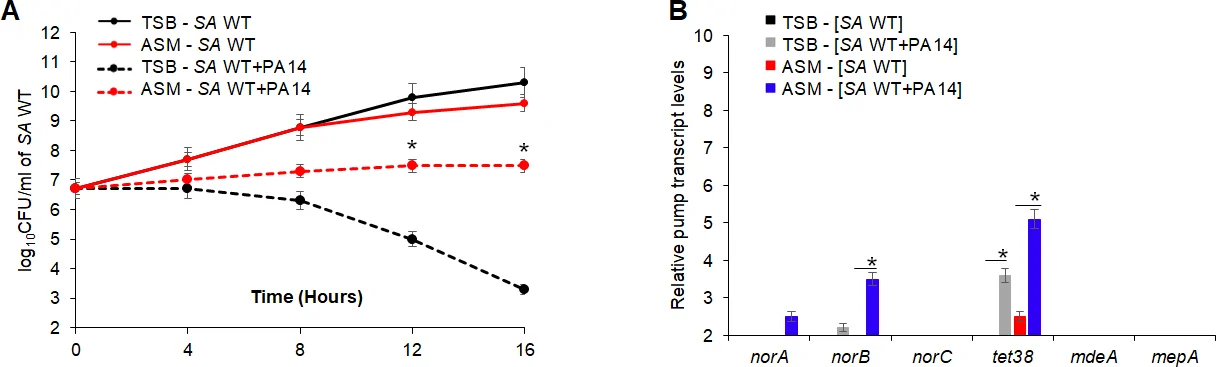
*S. aureus* response to *P. aeruginosa* in ASM medium. (**A**) Growth of the *SA* wild-type reference strains RN6390 and Newman (collectively referred to as *SA* WT). Each strain was analyzed independently following growth alone or in co-culture with PA14 in TSB or ASM for 16 h. Both strains exhibited comparable growth responses; therefore, they are presented collectively as the *SA* WT phenotype. The error bars represent the means of (log_10_ CFU/mL ± SD) for each growth curve. Assays were carried out with both RN6390 and Newman, yielding similar results. Differences (*) in the number of *SA* WT survivors following co-culture with PA14 in ASM vs in TSB were statistically significant as determined by ANOVA (*P* < 0.05). *SA* WT, RN6390 or Newman; PA14, *PA* reference strain. (**B**) Induction of MDR efflux pump genes in the *SA* wild-type reference strains RN6390 and Newman. Each strain was analyzed independently following growth in TSB or ASM, with or without PA14, and exhibited comparable transcriptional responses. Relative transcript levels are expressed as fold change compared with the corresponding strain grown in TSB. Error bars represent the mean fold change ± SEM from three independent biological experiments. Statistical significance (*) was determined by ANOVA (*P* < 0.05). *SA* WT, *S. aureus* RN6390 or Newman. [*SA* WT + PA14], co-culture with PA14.

The experiments shown in Fig. 1 were performed independently using the two *SA* wild-type reference strains RN6390 and Newmian (Table 1), which exhibited comparable responses under all experimental conditions. For clarity, these strains are collectively referred to as *SA* WT. Compared with growth in trypticase soy broth, *SA* WT reached a slightly lower cell density in ASM after 16 h; however, this difference was not statistically significant (Fig. 1A). ASM was used to model key features of the CF airway environment (37, 38). In contrast, during co-culture with PA14, *SA* maintained significantly greater survival in ASM than in TSB, sustaining a constant growth level of ~7-log_10_ CFU/mL compared with 3.3-log_10_ CFU/mL in TSB at 16 h (Fig. 1A), indicating that the CF-like environment altered the outcome of the polymicrobial interaction. Under the same conditions, PA14 growth was not significantly affected by co-culture with *SA* WT (Fig. S1).

**TABLE 1:** Bacterial strains, plasmids, and primers used in this study

Strains, plasmids, and primers	Genotypes or relevant characteristic(s)	Reference or source
*S. aureus*		
RN6390	wild type	(33)
Newman	MSSA, reference strain	(32)
*ΔnorA*	*norA* mutant	(32)
*ΔnorB*	*norB* mutant	(39)
*Δtet38*	*tet3*8 mutant	(32)
RN(pLI50)	RN6390 with empty plasmid, Cm^R^	(31)
RN(NorA)	RN6390(pLI50-*norA),* NorA*-*overexpressor, Cm^R^	(34)
RN(NorB)	RN6390(pLI50-*norB*), NorB-overexpressor, Cm^R^	(34)
RN(Tet38)	RN6390(pLI50-*tet38*), Tet38-overexpressor, Cm^R^	(34)
Non-CF *SA*	20 non-CF clinical isolates, designated SA-1 through SA-20	(40)
SA1, SA4, and SA5	Representative non-CF *SA* isolates (SA1, SA4, SA5) selected from the 20 non-CF clinical isolates^*a*^	(40)
CF-*SA*	30 clinical *SA* isolates recovered from people with cystic fibrosis (pwCF) at the MGH CF Center	This study, (32)
CF5, CF10, and CF50	Representative paired CF-*SA/PA* isolates selected from the 30 CF-*SA* clinical isolates^*a*^	This study
CF19	Representative CF-*SA* isolated alone selected from the 30 clinical CF-*SA* isolates^*a*^	This study
*P. aeruginosa*		
PA14	Reference strain, virulent burn wound isolate	(32)
PA3	Co-isolated with CF5	This study
PA7	Co-isolated with CF10	This study
PA16	Co-isolated with CF50	This study
*phzA-G*	PA14 mutant	(27)
*pqsA*	PA14 mutant	(41)
*pqsL*	PA14 mutant	(42)
Plasmid		
pLI50	Shuttle plasmid *E. coli–S. aureus*, Cm^R^	(34)
Primers (qRT-PCR)		
*gmk*	Forward 5’TCAGGACCATCTGGAGTAGGTAAAG 3’	(32)
	Reverse 5’CAAATGCGTGAAGGTGAAGTTGATG 3’	
*norA*	Forward 5’TGGCCACAATTTTTCGGTAT 3’	(32)
	Reverse 5’CTTTGGCTACATGTCAGCGA 3’	
*norB*	Forward 5' GCTACACCATCAACAGATACAGCAA 3’	(39)
	Reverse 5' ACTCAATGCGACGCCAAA 3’	
*norC*	Forward 5' TGGGTTGGAGATGGATTTTC 3’	(40)
	Reverse 5’ ACAATTAGCCCTGCAACGTC 3’	
*tet38*	Forward 5’ATCGTAGTATTTACGTTGCC 3’	(32)
	Reverse 5’GGCTTAATTCTAGTGGCAAC 3’	
*mdeA*	Forward 5’ CCAGAAATGAATGCACATGG 3’	This study
	Reverse 5’ CAGCGTGATAACCATGCAAC 3’	
*mepA*	Forward 5’ GGCAAATAAAGGCCGTATGA 3’	This study
	Reverse 5’ CAGTCGCTTGAAGCATACCA 3’	

^*a*^ Representative isolates were selected from the complete clinical collections for functional assays. Unless otherwise indicated, transcriptional analyses were performed using the full clinical isolate collections.

We determined the transcript levels of *SA* efflux pump genes following growth of *SA* WT alone in TSB and ASM (OD_600_ ~0.5–0.6) and found that *tet38* was slightly induced in ASM (2.5-fold increase) compared with other multidrug (MDR) efflux pumps (*norA, norB, norC, mdeA*, and *mepA*) and compared with *SA* WT alone in TSB (Fig. 1B).

In TSB, compared with *SA* WT alone, the *norB* transcript level increased 2.2-fold and *tet38* increased 3.6-fold in *SA* WT co-cultured with PA14. In ASM, compared with *SA* WT alone, *norA* increased 2.5-fold, *norB* increased 3.5-fold, and *tet38* increased from 2.5-fold to 5.1-fold in *SA* WT co-cultured with PA14 (Fig. 1B).

These results showed that co-culture of *SA* WT with PA14 in ASM selectively reprogrammed *SA* transcriptional responses. The CF-like environment provided by ASM enhanced *tet38* and *norB* expression and improved *SA* survival during coexistence with *PA*. Because ASM is a complex medium, our data do not identify the specific component(s) responsible for these responses.

### *P. aeruginosa* metabolites influence *S. aureus* survival and efflux pump activation

We investigated whether secreted metabolites of PA14, such as phenazines, HHQ, PQS, HQNO, and siderophores, played roles in the activation of the *SA* WT (RN6390 or Newman) efflux pump in ASM.

**Fig 2:**
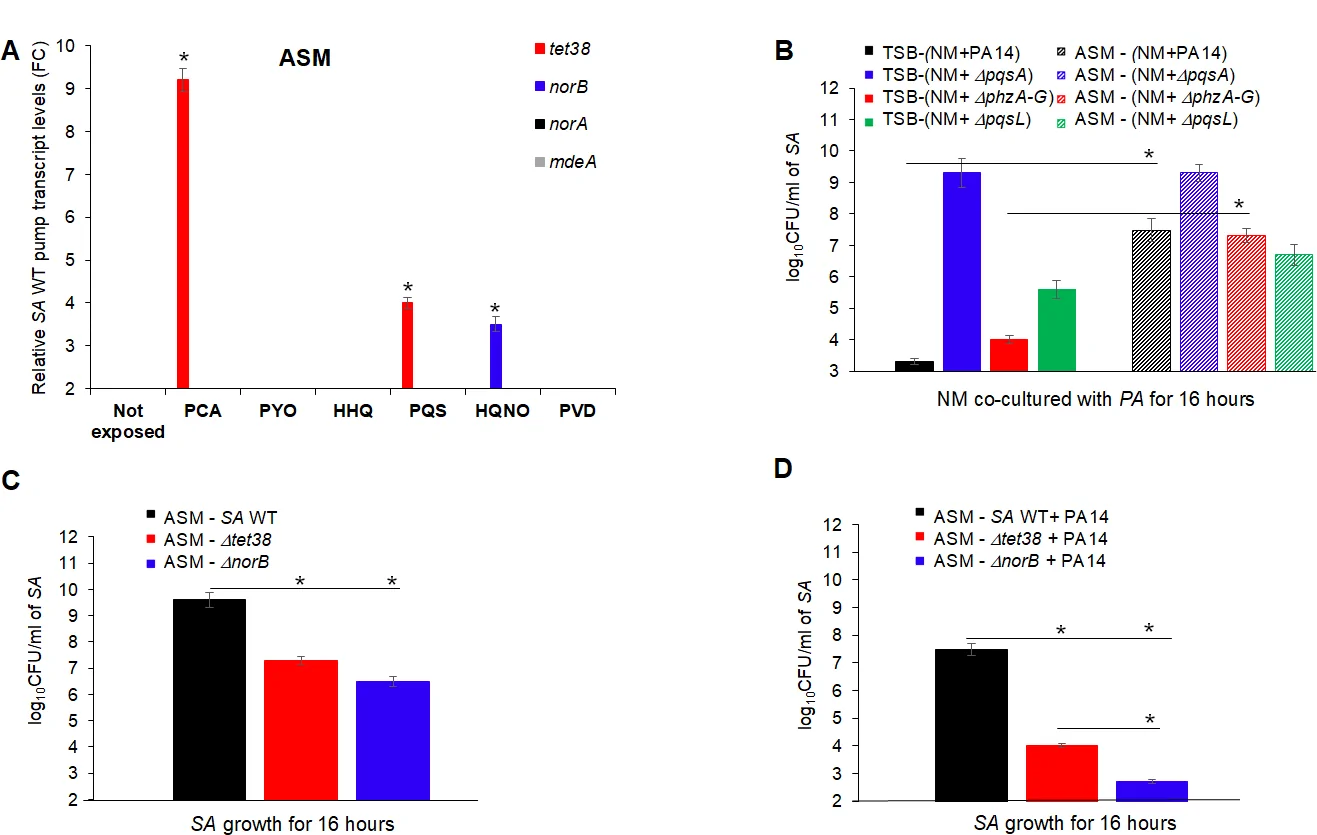
Roles of Tet38 and NorB in *S. aureus* survival under stress conditions. (**A**) Induction of *tet38* and *norB* by *P. aeruginosa* (*PA*) metabolites. Relative transcript levels of MDR efflux pump genes of *SA* WT, exposed to 0.5× MIC of *PA* metabolites in ASM, were expressed as the fold change (FC) in pump gene transcripts of *SA* WT (+*PA* metabolites) versus non-exposed. The assays were repeated three times with three different biological samples. The error bars represent the means of FC ± SEM for each assay. Differences (*) in the FC of *tet38* or *norB* of *SA* WT exposed to *PA* molecules versus *SA* WT non-exposed were statistically significant as determined by ANOVA (*P* < 0.05). (**B**) *SA* WT Newman co-cultures with PA14 and its mutants in TSB and ASM. The bar graphs represent the CFU/mL of Newman (NM) in co-culture with PA14 or PA14 mutants (*ΔpqsA, ΔpqsL, ΔphzA-G*) grown in TSB or ASM for 16 h. Differences (*) in NM survivors (+PA14) and NM survivors (+*ΔphzA-G*) in ASM vs in TSB were statistically significant as determined by ANOVA (*P* < 0.05). (**C**) Mutants *Δtet38* and *ΔnorB* growth curves in ASM. The bar graphs show the CFU/mL of *SA* WT (RN6390), mutants *Δtet38* and *ΔnorB,* grown in ASM for 16 h. Differences (*) between mutant *Δtet38* or *ΔnorB* and *SA* WT, grown in ASM, were statistically significant as determined by ANOVA (*P < 0.05*). Notes: *Δtet38* or *ΔnorB* are isogenic mutants of RN6390. (**D**) *SA* WT, mutants *Δtet38* and *ΔnorB*, co-culture with PA14 in ASM medium. The bar graphs represent the CFU/mL of *SA* WT (RN6390), *Δtet38*, and *ΔnorB* grown in ASM for 16 h. Differences (*) between mutant *Δtet38* or *ΔnorB* (+PA14) and *SA* WT (+PA14), grown in ASM, were statistically significant as determined by ANOVA (*P* < 0.05). All assays in panels **B–D** were repeated three times with three different biological samples. The error bars represent the means of (log_10_ CFU/mL ± SD) for each *SA* WT (±PA14 or *PA* mutants) growth assay. *SA* WT, *S. aureus* RN6390; (+ *PA*), *SA* co-cultures with *PA*.

We found that, in ASM, 0.5× MIC PCA (160 µg/mL; MIC_PCA_ = 320 µg/mL) and 0.5× MIC_PQS_ (2.5 µg/mL; MIC_PQS_ = 5 µg/mL) increased *tet38* transcript levels 9.2-fold and 4-fold, respectively, after *SA* WT was exposed to *PA* metabolites for 3 h, as previously reported (32, 33). We also found that 0.5× MIC HQNO (2-heptyl-4-hydroxyquinoline N-oxide) (4.0 µg/mL; MIC_HQNO_ = 8.0 µg/mL) induced *norB* expression (~3.5 fold increase) (Fig. 2A), confirming that *PA* phenazine (PCA), PQS, and HQNO were linked to upregulation of the *SA* WT efflux pumps *tet38* and *norB. SA* WT RN6390 and Newman showed similar results in these assays.

To assess the role of PCA, PQS, and HQNO in the *SA* WT response to *PA* killing in ASM compared with TSB, we performed co-culture assays of Newman (NM) with PA14, PA14 mutants *ΔphzA-G* (lack of phenazines) (27), *ΔpqsA* (lack of quinolines), and *ΔpqsL* (lack of HQNO) (28). In ASM, we found that NM grew better in a co-culture with mutant *ΔpqsA* than with PA14 and other mutants (Fig. 2B), suggesting that quinolines are the major factor in the anti-staphylococcal activity of PA14 in TSB.

Since *tet38* and *norB* were the efflux pumps most strongly associated with the *SA* response to ASM ± *PA*, we investigated the survival of the *Δtet38* and *ΔnorB* mutants (RN6390 background) in ASM during monoculture and co-culture with PA14. Both mutants exhibited impaired growth in ASM monoculture compared with the wild-type strain (RN6390) (Fig. 2C), demonstrating that Tet38 and NorB contribute to *SA* fitness under CF-like conditions independently of *PA*. After 16 h, *Δtet38* reached approximately 7.3 log10 CFU/mL and Δ*norB* approximately 6.5 log10 CFU/mL, compared with ~9.6 log10 CFU/mL for the wild-type strain. Consistent with our previous study (32), these findings confirm the contribution of Tet38 to survival in ASM and extend this role to NorB.

During co-culture with PA14, the survival defects of both mutants were further exacerbated (Fig. 2D), indicating that Tet38 and NorB also promote adaptation to polymicrobial stress. Together, these findings demonstrate that Tet38 and NorB contribute to *SA* fitness under both the nutritional constraints imposed by ASM and the additional stress associated with coexistence with *PA*.

### Tet38 and NorB are the dominant efflux pumps in *S. aureus* isolated from pwCF

We determined the transcript levels of *tet38, norA, norB, mdeA,* and *mepA* in 50 clinical *SA* isolates, comprising 30 CF isolates (18 CF-*SA* isolates recovered without *PA* and 12 CF-*SA/PA* isolates recovered from patients co-infected with *PA*) and 20 non-CF isolates, following growth for 24 h in TSB. A 3-fold or greater increase in efflux pump expression relative to *SA* Newman was considered significant, as established in previous studies (29, 43).

**Fig 3:**
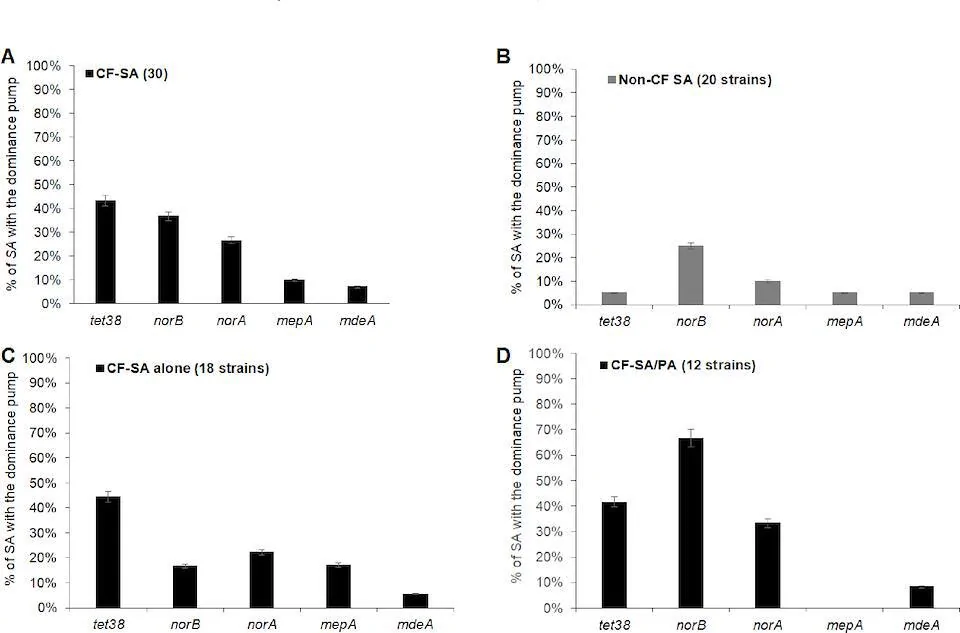
Hierarchical distribution of MDR efflux pumps of CF-associated *S. aureus*. (**A and B**) Distribution of dominant efflux pumps across clinical isolates. The percentage of isolates in which each efflux pump (*tet38, norB, norA, mepA,* and *mdeA*) was the most highly expressed (≥3-fold relative to the reference strain Newman) is shown for CF-*SA* (30 strains) (**A**) and non-CF *SA* (20 strains) (**B**). (**C and D**) Stratification of CF-*SA* based on polymicrobial context. CF-*SA* isolated alone (18 strains) preferentially exhibited *tet38* dominance (**C**), whereas CF-*SA* co-isolated with *PA* (12 strains) more frequently exhibited *norB* dominance while retaining substantial *tet38* representation (**D**). Percentages were calculated within each group based on the number of isolates in which a given pump showed the highest level of expression. All assays in this figure were repeated three times with three different biological samples. The error bars represent the means of (% ± SD) for each group.

Among the CF collection, five CF-*SA* isolates and five of the twelve CF-*SA/PA* isolates exhibited concurrent overexpression of two or more efflux pumps. Because the present study focused on the hierarchy of individual MDR efflux pump utilization, these co-overexpression patterns were not analyzed further. Table 2 and Fig. 3 show the percentage and distribution of efflux pump overexpressors among the 30 CF isolates and 20 non-CF isolates (Fig. 3A and B). Among the CF-*SA* isolates recovered without *PA*, 44% overexpressed *tet38* (Fig. 3C), whereas 67% of the CF-*SA/PA* isolates overexpressed *norB* (Fig. 3D). Overall, among the 30 CF isolates, 8 (26.7%) overexpressed *norA*, and five of these eight isolates (62.5%) carried *norA* promoter mutations (CAAT/ACAA/CTAT) (29, 32, 43). In contrast, among the 20 non-CF isolates, 25% overexpressed *norB*, 10% overexpressed *norA* (without *norA* promoter mutations), and 5% each overexpressed *tet38, mepA,* and *mdeA* (Tables 2 and 3).

**TABLE 2:** Percentage and distribution of efflux pump-overexpressing *S. aureus^a^*

Efflux pump-overexpressing	CF strains		non-CF strains
	CF-*SA*	CF-*SA/PA*	*SA*
	*n* (%)	*n* (%)	*n* (%)
*tet38*-overexpressing	8 (44)	5 (45)	1 (5)
*norB*-overexpressing	3 (17)	8 (67)	5 (25)
*norA*-overexpressing	4 (22)	4 (33)	2 (10)
*norA-*overexpressing isolates^*b*^	CF2, CF4, CF11, CF19	CF5, CF6, CF10, CF12	SA1, SA3
*mepA*-overexpressing	3 (17)	0 (0)	1 (5)
*mdeA*-overexpressing	1 (6)	1 (8)	1 (5)
Total isolates, *n* (%)	18 (60)	12 (40)	20 (100)
Total CF-*SA* + CF-*SA/PA*, *n* (%)	30 (100)		20 (100)

^*a*^ n, number of isolates. CF-*SA*, *SA* isolated alone; CF-*SA/PA*, *SA* co-isolated with *PA*. All non-CF isolates are designated SA-1 through SA-20 (20 isolates).

^*b*^ The isolate IDs listed correspond to strains exhibiting ≥3-fold *norA* overexpression relative to Newman. Representative non-CF isolates (SA-1, SA-4, and SA-5) were selected for growth curve assays in ASM (Fig. 4). Representative CF isolates (CF5, CF10, CF50, and CF19) were selected for the functional assays described in Fig. 4 and 5.

**Fig 4:**
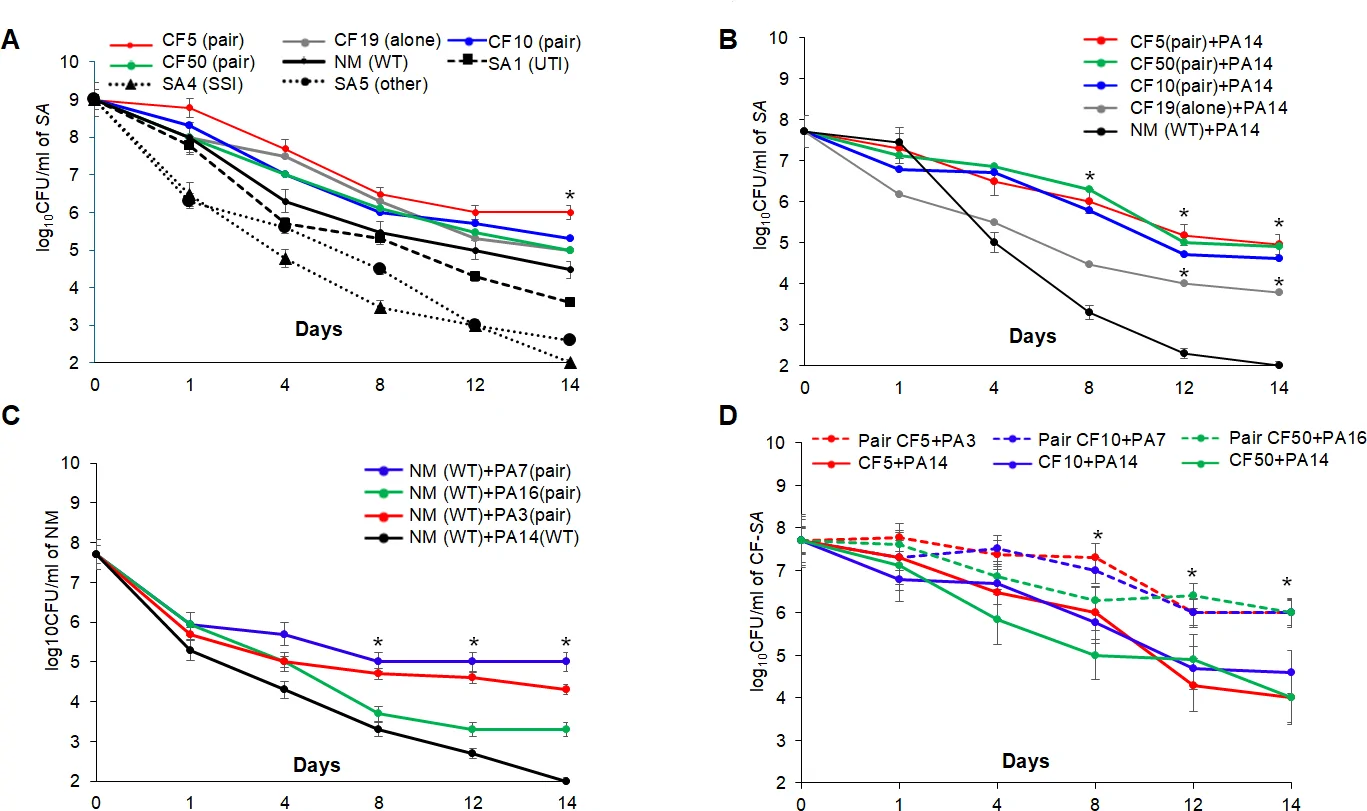
Fitness of CF-*SA* in ASM and with or without *PA*. (**A**) Monoculture of *SA* in ASM. NM WT (Newman), CF-*SA* (CF5, CF10, CF50, CF19), and non-CF *SA* (SA1, SA4, SA5) were cultured in ASM for 14 days. Differences (*) in log_10_ CFU/mL between CF-*SA* and non-CF *SA* in ASM at day 14 were statistically significant as determined by ANOVA (*P* < 0.05). CF5 (pair), CF10 (pair), CF50 (pair), CF-*SA* co-isolated with *PA*; CF19 (alone), CF-*SA* isolated alone; SA1, SA4, SA5, *S. aureus* from various infection sites; UTI, urinary tract infection; SSI, skin and soft tissue infection. (**B**) CF-*SA* strains in co-culture with PA14. NM (WT), CF5 (pair), CF10 (pair), CF50 (pair), and CF19 (alone) were co-cultured with PA14 in ASM for 14 days. Differences (*) in log_10_ CFU/mL of *SA* survivors between CF-*SA* and NM at 8, 12, and 14 days were statistically significant as determined by ANOVA (*P* < 0.05). (**C**) CF-*PA* strains of the pairs *SA/PA* in co-culture with Newman. NM (WT) was co-cultured with PA14, PA3 (pair), PA7 (pair), and PA16 (pair), in ASM for 14 days. Differences (*) in log_10_ CFU/mL of NM (WT) survivors after co-culture with PA3 (pair) and PA7 (pair) vs PA14 at 8, 12, and 14 days were statistically significant as determined by ANOVA (*P < 0.05*). (**D**) CF-*SA* strains co-culture with the corresponding *PA*. CF-*SA* of the pairs were co-cultured with the corresponding *PA* or PA14 in ASM for 14 days. Differences (*) in log_10_ CFU/mL of CF-*SA* (pair) in co-culture with *PA* (pair) vs in co-culture with PA14 at 8, 12, and 14 days were statistically significant as determined by ANOVA (*P* < 0.05). CF5/PA3, CF10/PA7, and CF50/PA16, pairs CF-*SA/PA; SA + PA*, co-culture assays. All assays in this figure were repeated three times with three different biological samples. The error bars represent the means of log_10_ CFU/mL ± SD for each assay.

**Fig 5:**
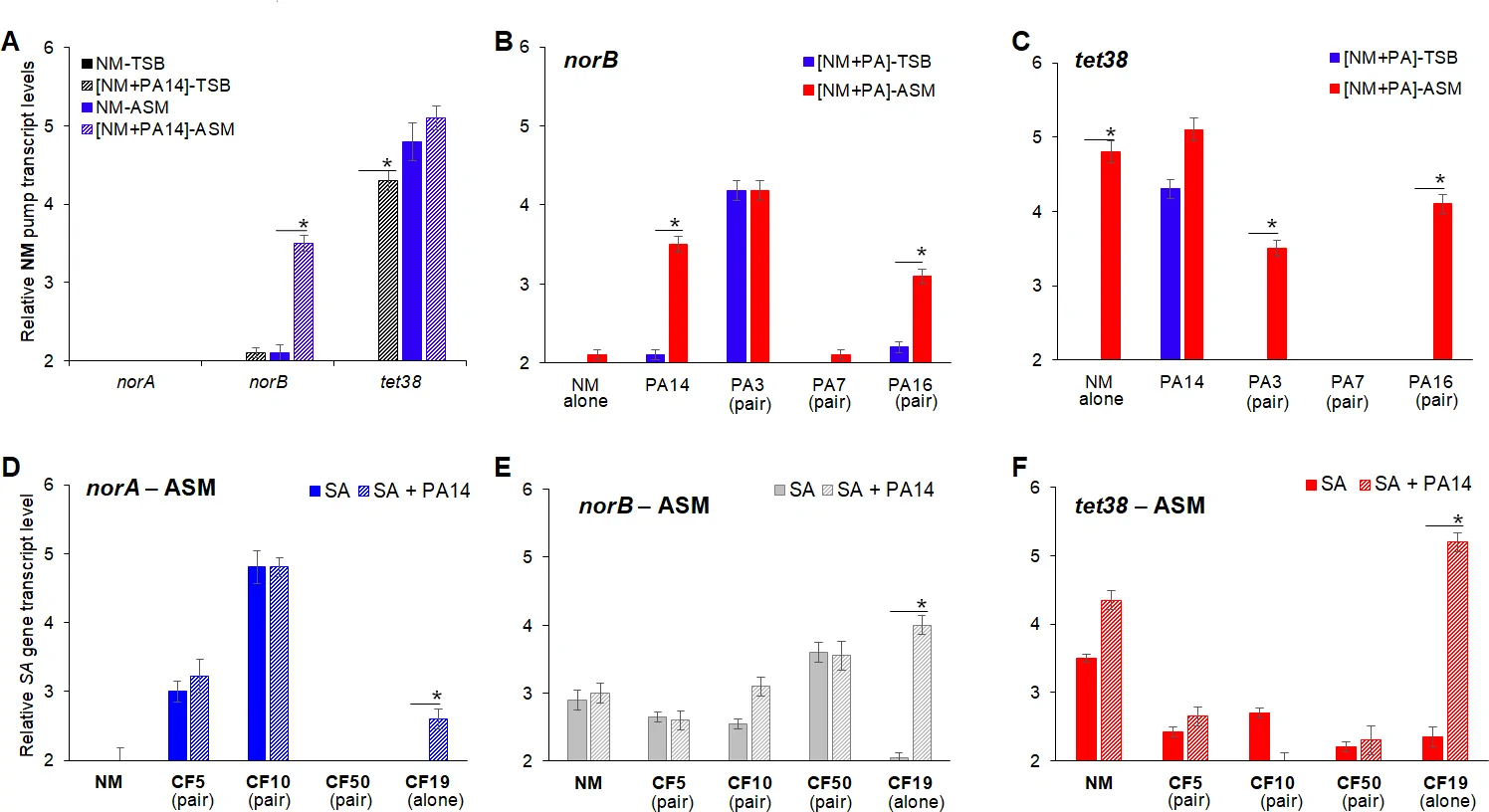
ASM medium and *P. aeruginosa* affect CF*-SA* efflux pump expression. (**A**) Induction of *S. aureus* MDR efflux pumps. The bar graphs represent the relative transcript levels of *norA, norB*, and *tet38* of NM (±PA14) grown in TSB or ASM. The efflux pump relative transcript levels were expressed as the fold change (FC) in pump gene transcripts of NM (+PA14) vs NM alone in TSB or ASM. Differences (*) in the FC of *norB* of NM (+PA14) vs NM alone in ASM and in the FC of *tet38* of NM (+PA14) vs NM alone in TSB were statistically significant as determined by ANOVA (*P* < 0.05). (**B and C**) Induction of *norB* and *tet38* of *S. aureus* in co-culture with PA14 or CF-*PA*. The bar graphs represent the transcript levels of *norB* (**B**) and *tet38* (**C**) of NM ± CF*-PA* or PA14 grown in TSB or ASM. The *norB* or *tet38* relative transcript levels were expressed as the FC in pump gene transcripts of NM (+*PA*) vs NM alone in each medium. Differences (*) in the *norB* FC of NM (+PA14) and NM (+PA16) in ASM vs TSB were statistically significant as determined by ANOVA (*P* < 0.05). Differences (*) in the *tet38* FC of NM alone, NM (+PA3), and NM (+PA16) in ASM vs TSB were statistically significant as determined by ANOVA (*P* < 0.05). NM + PA, co-culture with *PA*. (**D–F**) Induction of *norA*, *norB,* and *tet38* of *S. aureus* in co-culture with PA14. The bar graphs represent the transcript levels of *norA* (**D**), *norB* (**E**), and *tet38* (**F**) of NM or CF-*SA* (±PA14) in ASM. The efflux pump relative transcript levels were expressed as the FC in pump gene transcripts of (*SA* + PA14) vs *SA* alone in ASM. Differences (*) in the *norA, norB,* and *tet38* FC of (CF19 + PA14) vs CF19 alone in ASM were statistically significant as determined by ANOVA (*P* < 0.05). All assays in this figure were repeated three times with three different biological samples. The error bars represent the means of FC ± SEM for each assay.

**TABLE 3:** Characteristics of 30 CF-associated *S. aureus* (CF-*SA*) clinical strains used in this study

CF-*SA*	Type of *SA*	*norA* ^*a*^	CF-*SA/PA^b^*	MIC of CIP	MIC of TETRA
Newman		WT		0.5	0.5
CF-1	MSSA	WT		1	1
**CF-2**	MSSA	CAAT		>128	4
CF-3	MSSA	WT		0.5	0.5
**CF-4**	MSSA	WT		32	16
CF-5	MSSA	WT	PA3	2	2
CF-6	MSSA	ACAA	PA5	>128	1
CF-7	MRSA	*polX*		32	0.5
CF-8	MSSA	*polX*		32	0.5
CF-9	MSSA	WT		128	1
CF-10	MSSA	CAAT	PA7	8	2
**CF-11**	MSSA	CAAT		>128	4
CF-12	MRSA	CAAT	PA9	>128	1
CF-13	MSSA	WT		16	0.5
CF-16	MSSA	WT		0.5	0.5
CF-17	MSSA	WT	PA10	32	1
**CF-19**	MSSA	WT		2	1
CF-20	MSSA	CAAT		>128	0.5
CF-21	MSSA	*polX*		0.5	1
CF-23	MSSA	CAAT	PA12	>128	8
CF-31	MRSA	*polX*	PA15	64	1
CF-38	MSSA	WT		0.5	2
CF-40	MSSA	WT		0.5	0.5
CF-41	MSSA	WT		0.5	0.5
CF-42	MRSA	WT		1	1
CF-43	MSSA	WT		0.5	1
CF-50	MSSA	*polX*	PA16	1	1
CF-64	MRSA	CTAT	PA19	>128	0.5
CF-66	MSSA	WT	PA20	1	1
CF-71	MSSA	WT	PA25	1	1
CF-72	MSSA	WT	PA27	0.5	0.5

Total CF-*SA* isolates	% CF-*SA* with MIC_CIP_ ≤ 2	% CF-*SA* with MIC_TETRA_ ≤ 2			
30	50% (15/30)	86.7% (26/30)			

^*a*^ *norA*, *norA* promoter region with or without mutation.

^*b*^ Length of coinfection *SA/PA*: 16 years (PA3, PA5, PA9, PA15, PA25, PA27); ~8–9 years (PA7, PA10, PA12, PA16, PA19, PA20). CF-*SA/PA*, co-isolated pairs; CIP, ciprofloxacin; TETRA, tetracycline. **CF2**,** CF4**,** CF11**, and** CF19**: *norA-*overexpressing CF-*SA* isolated alone (Table 2). CF5, CF6, CF10, and CF12: *norA-*overexpressing CF-*SA* co-isolated with *PA* (Table 2).

Taken together, the CF-*SA* pump distribution showed a hierarchical dominance for Tet38 as the primary pump and NorB as the secondary dominant pump, compared to the conventional pump distribution of the non-CF *SA* isolated from other infection sites, following exposure to antibiotics (40). All CF-*SA* carried WT promoter regions and structural genes for *tet38* and *norB.* These data showed that CF-*SA* isolated alone preferentially overexpressed *tet38* (44%), and while CF-*SA* co-isolated with *PA* more frequently overexpressed *norB* (67%), they still preserved a significant proportion of *tet38* overexpression (42%).

### CF-*SA* co-isolated with *PA* are more adapted to the polymicrobial CF-like environment

We tested the fitness of CF-*SA* grown in ASM with and without *PA* over 14 days. We compared the survival of clinical strains isolated alone from pwCF (CF19), others co-isolated with *PA* (pairs CF5/PA3, CF10/PA7, and CF50/PA16), isolates from non-CF patients (SA1, SA4, and SA5) (Tables 1 and 2) (40), and reference strain NM. All non-CF *SA* strains survived poorly in the ASM medium, with ~1 to 2-log CFU/mL less than NM. In contrast, all CF-*SA* grew better than NM in ASM, with ~1 to 2-log CFU/mL more survivors after 14 days of growth (Fig. 4A).

In a co-culture with strain PA14 for 14 days, all CF-*SA* of the *SA/PA* pairs grew better than NM in ASM (3-log CFU/mL more survivors), while CF19 (alone) survived slightly less well than the *SA* pairs (~1-log CFU/mL fewer survivors), still better than NM (~1-log CFU/mL more survivors) (Fig. 4B). To investigate whether CF-*PA* of the *SA/PA* pairs were less virulent than PA14, we determined the CFU/mL of NM in co-culture with PA3, PA7, and PA16 in ASM medium and compared with co-culture with PA14. After 14 days, we found that NM exposed to CF-*PA* survived better than NM exposed to PA14, with a variation of 1–3-log_10_ CFU/mL (Fig. 4C), in keeping with the known robust virulence properties of PA14 (44).

To investigate whether the CF-*SA* of the pairs survived better in a co-culture with their corresponding *PA* strains than with PA14, we determined the *SA* CFU/mL of CF5 (+PA3), CF10 (+PA7), and CF50 (+PA16) and compared them to those of CF5 (+PA14), CF10 (+PA14), and CF50 (+PA14). We found that the survivors of *SA* CF5, CF10, and CF50 co-cultured with their corresponding *PA* were ~2-log CFU/mL higher than those co-cultured with PA14 (Fig. 4D). In addition, CF-*SA* survivors of the pair *SA/PA* were higher than those of NM in co-culture with CF-*PA* (PA3, PA7, and PA16) or with PA14 in ASM for 14 days (Fig. 4C and D). Furthermore, we found that all *PA* strains of the pairs CF-*SA/PA* and PA14 grew well after 14 days in ASM medium (Fig. S1).

### CF-*SA* activates the transcription of *tet38* and *norB* in response to *PA* in ASM medium

We investigated whether CF-*PA* triggered increased expression of *SA* efflux pumps. We carried out co-cultures of NM with PA14 and the 3 CF-*PA* in TSB and ASM for 3 h, then measured the transcript levels of *norA, norB*, and *tet38*.

We found that, in ASM, NM (+PA14) relative to NM alone showed a 3.5-fold increase in *norB* transcript level, while *tet38* showed a similar increase (~5-5.3-fold) for NM ± PA14 (Fig. 5A). In TSB, NM (+PA14) relative to NM alone showed a 4.8-fold increase in *tet38* transcript level, while *norA* showed a less than 2-fold increase when co-cultured with PA14 (Fig. 5A).

Compared to NM alone, a co-culture of NM in ASM with PA14 and clinical *PA* isolates resulted in 3- to 4-fold increases in *norB* and *tet38* transcript levels with PA3 and PA16, but minimal change with PA7. Lesser effects of *PA* strains were seen in TSB relative to ASM, except for similar induction of *norB* in co-culture with PA3 (Fig. 5B and C).

We next investigated whether PA14 could induce *norA, norB*, and *tet38* of CF-*SA* clinical isolates. We carried out co-cultures of CF19 (alone, WT *norA* promoter), CF5 (paired with PA3, WT *norA* promoter), CF10 (paired with PA7, CAAT *norA* promoter), and CF50 (paired with PA16, *polX* insertion-*norA* promoter) (Table 3) with PA14 in ASM medium for 3 h, then measured the transcript levels of CF-*SA* pumps. We observed that CF5 and CF10 showed a ~3-fold and 5-fold increase in *norA* transcript levels in mono- and co-culture with PA14, respectively. CF19 only showed a 2.6-fold increase in *norA* when co-cultured with PA14 in ASM (Fig. 5D). Notably, CF19 in co-culture with PA14 also showed a significant increase in *norB* and *tet38* (>2-fold) compared to CF19 in monoculture. CF-*SA* CF5, CF10, and CF50 showed increases of ~2 to 4-fold in *norB* and *tet38* in ASM, and a co-culture with PA14 had no significant additional effect on the transcript levels (Fig. 5E and F).

### MDR efflux pumps cause a low-level resistance to antibiotics in CF-*SA*

For comparison, we determined the MICs of antibiotics of CF19, CF5, CF10, and CF50 and compared them to those of *SA* Newman (NM). We found that CF-*SA* exhibited a low level of antibiotic resistance. CF19 showed a 4-fold increase in the MICs of ciprofloxacin (CIP) and a 2-fold increase in the MIC of tetracycline (TETRA). CF5 showed a 4-fold increase in the MICs of CIP and TETRA and a 2-fold increase in the MICs of levofloxacin (LVX) and doxycycline (DOXY). CF10 showed an 8-fold increase in the MIC of CIP, a 4-fold increase in the MIC of TETRA, and a 2-fold increase in the MICs of LVX and DOXY. CF50 showed a 4-fold increase in the MIC of CIP and a 2-fold increase in the MICs of TETRA and DOXY (Table 4). We performed DNA sequencing of the quinolone-resistance-determining region of the *gyrA, gyrB, parC*, and *parE* genes, which are the target genes of quinolones, and found no known mutations causing high-level resistance to CIP or LVX in CF5, CF10, CF50, and CF19 (45). We analyzed the MICs of CIP and TETRA of the 30 CF-*SA* strains randomly selected for this study, including CF5, CF10, CF50, and CF19, and found that 50% (15 out of 30 strains) and 86.7% (26 out of 30 strains) of CF-*SA*s exhibited a MIC of CIP or TETRA between 0.5 and 2 µg/mL (Table 3). Thus, the resistance levels of most CF-*SA* strains were similar to those of previously reported pump-related resistance phenotypes associated with *norA, norB*, and *tet38* (40, 43, 46).

**TABLE 4:** *S. aureus* MICs of antibiotics^*a*^

Strain	MICs of antibiotics				
	CIP	LVX	TETRA	DOXY	NAF
*S. aureus*					
RN6390	0.5	0.5	0.5	0.5	0.5
RN(pLI50)	0.5	0.5	0.5	0.5	0.5
*ΔnorA*	0.25	0.5	0.5	0.5	0.5
RN(NorA)	2	1	0.5	0.5	0.5
*ΔnorB*	0.5	0.5	0.5	0.5	0.5
RN(NorB)	2	1	0.5	0.5	0.5
*Δtet38*	0.5	0.5	0.25	0.25	0.5
RN(Tet38)	0.5	0.5	2	2	0.5
CF-*SA* strains					
Newman	0.5	0.5	0.5	0.5	0.5
CF19	2	0.5	1	0.5	0.5
CF5	2	1	2	1	0.5
CF10	4	1	2	1	0.5
CF50	2	0.5	1	1	0.5

^*a*^ CIP, ciprofloxacin; LVX, levofloxacin; TETRA, tetracycline; DOXY, doxycycline; NAF, nafcillin. RN6390, *S. aureus* wild-type reference strain; RN(pLI50), RN6390 transformed with the empty plasmid pLI50; RN(NorA), RN(NorB), RN(Tet38), RN6390 overexpressing efflux pumps. Newman, *S. aureus* reference MSSA strain.

## DISCUSSION

Persistence of *SA* in CF airways remains difficult to reconcile with its frequent *in vitro* susceptibility to antibiotics. In our previous work and that of others, many CF-*SA* isolates exhibited MIC values within the susceptible range despite clear evidence of persistence in the airway environment (32, 47). These observations support the concept that *SA* persistence in CF is not solely dependent on high-level resistance but can occur in isolates that remain susceptible or have low-level resistance by standard MIC criteria. In this study, we showed that *SA* survival in a CF-like environment was supported by selective reprogramming of MDR efflux pumps in response to nutritional and polymicrobial stress rather than by acquisition of high-level resistance. Using ASM and co-culture with *PA*, we identified Tet38 and NorB as key components of this adaptive response.

ASM medium (37) imposed a physiologically restrictive environment for *SA* while triggering a highly specific transcriptional response characterized by preferential induction of *tet38*. Other efflux pumps showed comparatively modest changes, indicating that *SA* does not activate a generalized MDR resistance program under conditions dictated by ASM. Instead, the data support a model in which efflux activity is selectively tuned to the CF-like conditions represented by ASM, with Tet38 representing a primary adaptive response that contributes to *SA* fitness. Consistent with this adaptive role, deletion of *tet38* resulted in a significant reduction in *SA* survival in ASM (Fig. 2C). Notably, this induction occurred in the absence of antibiotics, indicating that it is elicited by one or more components of the CF-like environment rather than by direct antibiotic exposure. Although ASM enhanced *SA* survival and selectively induced *tet38* expression, the present study does not distinguish whether these responses reflect altered nutrient availability, reduced antagonism by *PA*, or other environmental properties of the CF-like medium. Because ASM contains multiple components that collectively mimic the CF airway environment, the observed phenotype is likely influenced by several interacting factors rather than a single constituent. Defining the relative contribution of individual components, including mucin, extracellular DNA, nutrient composition, microbial metabolites, and host-derived factors, will require future investigation.

The presence of *PA* further modified this response. In co-culture, *SA* maintained *tet38* expression while increasing transcription of *norB* and, to a lesser extent, *norA* and *mdeA*, consistent with a redistribution of efflux activity under polymicrobial stress. Previous experiments with purified *PA* metabolites and mutant strains indicated that quinolines and phenazines contributed to this effect, linking metabolite-mediated stress to efflux induction (32). Among these pumps, Tet38 and NorB remained functionally important, as their deletions reduced *SA* fitness in both ASM and co-culture conditions. Together, these findings indicated that *SA* integrated environmental and interspecies signals to dynamically adjust efflux pump utilization. These findings prompted us to place our observations within the broader context of polymicrobial-driven adaptation.

Our findings are consistent with and extend prior work demonstrating that *PA* can broadly reprogram *SA* physiology during polymicrobial interaction. Transcriptomic analyses have shown that exposure to *PA* induces widespread changes in *SA* gene expression across pathways involved in metabolism, antibiotic response, and host interaction, highlighting the central role of interspecies signaling in shaping bacterial phenotypes (17). Complementing these global observations, mechanistic studies have established that Tet38 is a tightly regulated efflux pump linked not only to antimicrobial tolerance but also to host-cell interaction and adaptive physiology (25, 32). Additional work has further demonstrated that *SA* undergoes metabolic and transcriptional adaptation in response to *PA*, including shifts associated with altered antibiotic susceptibility and persistence phenotypes (35, 36). At the clinical level, longitudinal analyses of CF airway infection further support the persistence of both organisms and the importance of their interaction in shaping disease progression (19).

Our results extend these observations by demonstrating that, within the MDR efflux systems examined in this study, adaptation to CF-like conditions is associated with a reproducible hierarchy of efflux pump utilization. Tet38 emerged as the predominant adaptive response under ASM conditions, whereas NorB became increasingly important during polymicrobial interaction with *PA*. Although our analysis focused on five major chromosomally encoded MDR efflux pumps rather than the global transcriptome, the reproducibility of this hierarchy across laboratory strains, mutant analyses, and clinical isolates supports its biological relevance.

Analysis of CF clinical isolates further supported the physiological relevance of this model. CF-*SA* isolates exhibited a non-random and hierarchical distribution of efflux pump expression, with enrichment of Tet38 and NorB compared to non-CF isolates. Specifically, Tet38 was the most frequently overexpressed pump (43%, 13 out of 30 CF-*SA*), followed by NorB (37%, 11 out of 30 CF-*SA*), whereas NorA, MepA, and MdeA were less commonly represented. In contrast, non-CF isolates displayed lower and more variable expression, with only a small fraction overexpressing *tet38*. This hierarchy was further shaped by polymicrobial context: CF-*SA* isolated alone preferentially overexpressed *tet38* (44%), whereas CF-*SA* co-isolated with *PA* showed a marked increase in *norB* expression (67%) while maintaining substantial *tet38* activity (42%).

Notably, NorA overexpression in CF-*SA* was frequently associated with promoter mutations, whereas *tet38* and *norB* remained genetically conserved, suggesting that their regulation is primarily transcriptional and environmentally driven. Although NorA has been extensively implicated in fluoroquinolone resistance and was a major focus of our previous work, it did not emerge as the dominant environmentally responsive efflux pump under the CF-like conditions examined here. This distinction likely reflects the different mechanisms governing NorA versus Tet38 and NorB expression. Consistent with our previous studies, a subset of CF isolates overexpressed *norA*, and most carried well-characterized *norA* promoter mutations associated with constitutive overexpression (32). Accordingly, NorA expression varied among clinical isolates and exhibited only limited or isolate-dependent induction following co-culture with *P. aeruginosa*. In contrast, *tet38* and *norB* were consistently induced by ASM and polymicrobial growth despite the absence of promoter or structural mutations, indicating that these pumps are regulated primarily by environmental cues rather than fixed genetic alterations. Moreover, clinical isolates with constitutively elevated NorA expression still demonstrated induction of Tet38 and NorB under CF-like conditions, suggesting that environmental adaptation is achieved through reprogramming of a conserved efflux network rather than by further increasing NorA expression. Together, these findings indicate that NorA contributes predominantly to strain-specific antibiotic resistance, whereas Tet38 and NorB represent the principal environmentally responsive efflux systems that facilitate adaptation to the CF airway and polymicrobial interactions.

Collectively, these findings suggest that chronic adaptation to the CF airway is associated with stable efflux configurations that differ from those typically associated with antibiotic exposure alone (43, 48). Consistent with this interpretation, CF isolates displayed only modest increases in MICs but demonstrated enhanced survival in ASM, alone or in the presence of *PA*. Efflux pumps contribute to low-level resistance phenotypes; however, their role in supporting survival under combined environmental and antimicrobial stress appears to be more significant. We also observed that clinical *PA* isolates were less inhibitory toward *SA* than the reference strain PA14 and that paired *SA* isolates exhibited improved survival with their corresponding *PA* partners. These findings are consistent with adaptation toward coexistence in chronic infection, in which interspecies interactions shift from acute antagonism to a more stable equilibrium (49). Within this context, efflux systems such as Tet38 and NorB may contribute to *SA* persistence by mitigating the impact of residual inhibitory factors. Although the duration of *SA–PA* co-infection ranged from 8 to 16 years in our cohort, we did not observe a clear relationship between the documented duration of co-infection and efflux pump expression, *norA* promoter genotype, or antibiotic susceptibility. The relatively small cohort and limited range of co-infection durations preclude definitive conclusions regarding temporal adaptation. Larger longitudinal studies will be required to determine whether prolonged polymicrobial colonization contributes to progressive remodeling of efflux regulation (Table S1).

Taken together, our results support a model in which conserved MDR efflux systems are differentially utilized during adaptation to the CF airway. Whereas NorA contributes primarily to strain-specific antibiotic resistance through constitutive genetic alterations, environmental conditions characteristic of the CF airway, together with polymicrobial interactions, selectively reprogram Tet38 and NorB expression to promote bacterial persistence without conferring high-level resistance. This framework helps explain why antibiotic-susceptible *SA* can persist during therapy and highlights the importance of studying antimicrobial responses in physiologically relevant conditions. Most importantly, our findings suggest that efflux-mediated tolerance emerges as a consequence of environmental adaptation rather than simply as a direct response to antibiotic exposure.

Future studies will be needed to identify the upstream regulatory networks and the specific CF airway cues that govern this efflux reprogramming and to determine whether disrupting these adaptive pathways can enhance antimicrobial efficacy. In this study, we used ASM to recapitulate key features of the CF airway, including nutrient complexity and the production of *PA* metabolites such as quinolines and phenazines (38). Although this model captures important aspects of the polymicrobial CF environment, it does not distinguish the relative contributions of individual environmental signals. Defining how specific factors, including mucin, extracellular DNA, nutrient limitation, microbial metabolites, and host-derived cues, coordinate efflux remodeling will be essential for understanding the mechanisms underlying *SA* persistence and for identifying new therapeutic strategies to limit chronic infection.

## MATERIALS AND METHODS

### Bacterial strains, ASM media, and growth conditions

Bacterial strains, plasmids, and primers used in this study are listed in Table 1. Non-CF *SA* was selected from a collection of clinical isolates from the KOISA study (Korean Invasive *Staphylococcus aureus* Infection Study Group) (40).

Ciprofloxacin, levofloxacin, tetracycline, doxycycline, nafcillin, PCA, PYO, PQS, 2-heptyl-4-quinolone (HHQ), 4-hydroxy-2-heptylquinoline (HQNO), pyoverdine (PVD), mucin from porcine gastric stomach, fish sperm DNA, L-amino acids, NaCl, KCl, egg yolk emulsion, and diethylenetriaminepentaacetic acid (DTPA) were purchased from Sigma-Aldrich (St. Louis, MO).

*SA* WT RN6390 containing plasmid pLI50 and efflux pump constructs were grown at 37°C under shaking in TSB medium supplemented with chloramphenicol at 10 µg/mL. All other bacteria were grown in TSB medium unless otherwise stated. The ASM medium was prepared as previously described by Kirchner et al. (37). The mannitol-salt agar (MSA) for *SA* selection and cetrimide agar for *PA* selection were purchased from Fisher Scientific (Pittsburgh, PA).

### CF clinical isolates collection

*SA* (CF-*SA*) and *PA* (CF-*PA*) from patient sputum and throat samples were collected following participant consent and assent when appropriate, at the Massachusetts General Hospital (MGH) Cystic Fibrosis Center (Institutional Review Board no. 2011P000620) over a period of 36 months, from February 2023 to February 2026. Patient characteristics and CFTR modulator use were extracted from medical records. Isolates were cultivated in trypticase soy broth (TSB), streaked out on TSA agar plates, and grown at 37°C for 24 h. All strains grew normally within 24 h.

### Antibiotic susceptibility assay

The MIC was determined by broth microdilution in Mueller-Hinton broth at 37°C for 24 h, as previously described (31). A log-phase culture of *SA* (OD_600_ = 0.5) grown in TSB media was diluted 100-fold and inoculated into microtiter plates (Fisher Scientific, Pittsburgh, PA) containing two-fold serial dilutions of ciprofloxacin, levofloxacin, tetracycline, doxycycline, or nafcillin, or *PA* molecules (PCA, PYO, HHQ, PQS, HQNO, and PVD). MIC was the lowest drug concentration that produced no visible turbidity after incubation at 37°C for 24 h.

### Quantitative real-time RT-PCR assay

The real-time RT-PCR assays were done as previously described (32). Total *SA* RNA was extracted from lysostaphin-treated cells using the RNeasy midi kit (Qiagen, Valencia, CA). cDNAs were synthesized using the Verso cDNA synthesis kit (Thermo Scientific, ABgene, Epsom, Surrey, United Kingdom), followed by real-time RT-PCR assays using EvaGreen dye and the CFX96 real-time system (Bio-Rad, Hercules, CA). Primers designed for the RT-PCR assays were synthesized at Eton Bioscience Inc. (Eton Bioscience, Boston, MA) and are listed in Table 1. The housekeeping gene *gmk* was used as an internal control. All samples were analyzed in triplicate, and expression levels were normalized against *gmk* gene expression, which remained unchanged following exposure to ASM ± *PA* or to 0.5× MIC of *PA* molecules.

### Growth curves of *S. aureus* and *P. aeruginosa* strains in TSB and ASM

*SA* (RN6390, Newman, *Δtet38* and *ΔnorB* mutants, CF-*SA*, non-CF *SA*) and *PA* (PA14, PA14 mutants *ΔpqsA, ΔpqsL*, *ΔphzA-G* mutants, CF-*PA*) were cultured overnight at 37°C in TSB medium. Then, 10^5^ CFU/mL of each culture was transferred into 10 mL of fresh TSB or ASM medium and then cultured at 37°C under shaking for 16 h. For a co-culture of *SA* with *PA*, the overnight bacterial cultures were diluted to an OD_600_ ~0.1 (~10^4^ CFU/mL), continued to grow for an additional 1–2 h, then *SA* and *PA* were mixed in equal volume (5 mL) in TSB or ASM and cultured at 37°C for 16 h. Bacterial samples were collected every 4 h, diluted, and plated on TSA agar for colony counts (CFU/mL) if *SA* was in monoculture, and on MSA if *SA* was co-cultured with *PA* (17, 32).

For a 14-day growth, single colonies of *SA* (Newman, CF5, CF10, CF50, CF19, SA1, SA4, and SA5) or *PA* (PA14, PA3, PA7, and PA16) were inoculated separately in 5 mL of ASM medium, then cultured at 37°C for 24 h (Day 1). *SA* and *PA* were harvested by centrifugation (5,000 × g, 10 min), then resuspended together in fresh ASM medium and continued to grow for another 24 h (Day 2). The process was repeated for 14 days with daily fresh medium exchange, and bacteria were collected every 4 days for colony counting in MSA. *PA* was plated on cetrimide agar for colony counts.

For qRT-PCR assays, *SA* WT (RN6390 or Newman) and CF-*SA*, in monocultures or co-cultures with *PA*, were grown in TSB (2 h) or ASM (3 h), then the bacteria were harvested and submitted to RNA extraction and qRT-PCR assays.

To determine the dominance of CF-*SA* efflux pumps, CF-*SA* and non-CF *SA* were initiated from overnight cultures and grown in TSB at 37°C for 24 h, then the bacteria were harvested for qRT-PCR assays to determine the transcript level of *SA* efflux pumps. CF-*SA* co-isolated with *PA* were cultured together with the corresponding CF-*PA* to maintain the biological settings.

### *PA* phenazine and quinoline exposure assays

We determined the MICs of PCA, PYO, HHQ, PQS, HQNO, and PVD of *SA* WT reference strains (RN6390 and Newman), then carried out the exposure assays using 0.5× MIC concentrations of *PA* molecules as previously described (32, 50). *SA* WT initiated from an overnight culture was grown in ASM until OD_600_ ~0.4, then 0.5× MIC of *PA* metabolites were added to separate *SA* WT cultures in ASM for 3 h at 37 °C under shaking. Bacterial pellets were harvested and submitted to qPCR assays to assess the transcript levels of *norA*, *norB, tet38*, and *mdeA*.

### Statistical analysis

The experiments were performed in triplicate with three biological samples. The data were expressed as a mean ± SD. The data were analyzed using one-way or two-way analysis of variance (ANOVA). The pairwise comparison was done with a *t*-test with Bonferroni adjustment to compare sample groups. The threshold for significance was set at a *P*-value < 0.05.

## SUPPLEMENTAL MATERIAL

Fig. S1 aac.01070-26-s0001.tif Growth of PA in monoculture and co-culture with SA in TSB and ASM.

Supplemental material aac.01070-26-s0002.pdf Supplemental figure legend.

Table S1 aac.01070-26-s0003.docx Clinical characteristics of paired CF *S. aureus*-*P. aeruginosa* isolates, including duration of documented co-infection, *norA* promoter genotype, and antibiotic susceptibility.

ASM does not own the copyrights to Supplemental Material that may be linked to, or accessed through, an article. The authors have granted ASM a non-exclusive, world-wide license to publish the Supplemental Material files. Please contact the corresponding author directly for reuse.
