# Supplementary material for: Efflux reprogramming promotes Staphylococcus aureus persistence with Pseudomonas aeruginosa in cystic fibrosis
Source: Antimicrob Agents Chemother. 2026 Oct 8;70(11):e01070-26. doi: 10.1128/aac.01070-26 (PMC13731171; doi:10.1128/aac.01070-26)
Supplement: Supplementary file 1 [file aac.01070-26-s0002.pdf]

Efflux Reprogramming Promotes *Staphylococcus aureus* Persistence with *Pseudomonas aeruginosa* in Cystic Fibrosis

Truong-Bolduc Q.C., Wang Y., Alvarez-Carcamo B., Steifman C., Lawton B.G., Yonker L.M., Hurley B.P., Rahme L.G., and Hooper D.C.

Supplemental Figure S1. Growth of *PA* in monoculture and co-culture with *SA* in TSB and ASM.

Top left: Growth of PA14 cultured alone or co-cultured with *SA* Newman (NM) or RN6390 (RN) in TSB or ASM for 14 days. No significant differences in PA14 growth were observed between monoculture and co-culture under either growth condition (ANOVA, $p > 0.05$).

Top right: Growth of PA14 and clinical CF-*PA* isolates (PA3, PA7, and PA16) cultured alone or co-cultured with Newman (NM) in ASM for 14 days.

Bottom panels: Growth of PA14 and the corresponding clinical CF-*PA* isolates (PA3, PA7, and PA16) cultured alone or co-cultured with representative CF-*SA* isolates CF5, CF10, and CF50, respectively, in ASM for 14 days. No significant differences in *P. aeruginosa* growth were observed between monoculture and co-culture in any experiment (ANOVA, $p > 0.05$).

CF5/PA3, CF10/PA7, and CF50/PA16 represent paired CF *SA/PA* clinical isolates.

NM, Newman; RN, RN6390.

All experiments were performed with three independent biological replicates. Data are presented as mean log10 CFU/ml ± SD.
