# Supplementary material for: Efflux reprogramming promotes Staphylococcus aureus persistence with Pseudomonas aeruginosa in cystic fibrosis
Source: Antimicrob Agents Chemother. 2026 Oct 8;70(11):e01070-26. doi: 10.1128/aac.01070-26 (PMC13731171; doi:10.1128/aac.01070-26)
Supplement: Supplementary file 2 [file aac.01070-26-s0003.docx]

**Efflux Reprogramming Promotes *Staphylococcus aureus* Persistence with *Pseudomonas aeruginosa* in Cystic Fibrosis**

Truong-Bolduc Q.C., Wang Y., Alvarez-Carcamo B., Steifman C., Lawton B.G., Yonker L.M., Hurley B.P., Rahme L.G., and Hooper D.C.

**Supplementary data**

**Supplementary Table S1.** Clinical characteristics of paired CF *S. aureus–P. aeruginosa* isolates, including duration of documented co-infection, *norA* promoter genotype, and antibiotic susceptibility.

|  | CF-*SA* | Type of *SA* | *norA* | CF-*SA/PA* | Coinfection (years) | CIP | TETRA |
| --- | --- | --- | --- | --- | --- | --- | --- |
|  | Newman |  | WT |  |  | 0.5 | 0.5 |
|  | CF-5 | MSSA | WT | PA3 | 16 | 2 | 2 |
|  | CF-6 | MSSA | ACAA | PA5 | 16 | >128 | 1 |
|  | CF-10 | MSSA | CAAT | PA7 | 9 | 8 | 2 |
|  | CF-12 | MRSA | CAAT | PA9 | 16 | >128 | 1 |
|  | CF-17 | MSSA | WT | PA10 | 9 | 32 | 1 |
|  | CF-23 | MSSA | CAAT | PA12 | 8 | >128 | 8 |
|  | CF-31 | MRSA | *polX* | PA15 | 15 | 64 | 1 |
|  | CF-50 | MSSA | *polX* | PA16 | 9 | 1 | 1 |
|  | CF-64 | MRSA | CTAT | PA19 | 9 | >128 | 0.5 |
|  | CF-66 | MSSA | WT | PA20 | 9 | 1 | 1 |
|  | CF-71 | MSSA | WT | PA25 | 16 | 1 | 1 |
|  | CF-72 | MSSA | WT | PA27 | 16 | 0.5 | 0.5 |

______________________________________________________________________________

**norA*, *norA* promoter region with or without mutation.

CF-*SA/PA*, co-isolated pairs; CIP, ciprofloxacin; TETRA, tetracycline.
